# Co-localization of clusters of TCR-regulated genes with TAD rearrangements

**DOI:** 10.1186/s12864-023-09693-8

**Published:** 2023-10-28

**Authors:** Galen F. Gao, Peng Li, Warren J. Leonard

**Affiliations:** Laboratory of Molecular Immunology and the Immunology Center, National Heart, Lung, and Blood Institute, National Institutes of Health, Bethesda, MD 20892-1674 USA

**Keywords:** TADs, Chromatin conformation, Epigenetics, Gene expression, Immunoinformatics, Multi-omics, CD4^+^ T cells, TCR activation

## Abstract

**Background:**

Gene expression has long been known to be influenced by the relative proximity of DNA regulatory elements. Topologically associating domains (TADs) are self-interacting genomic regions involved in regulating gene expression by controlling the proximity of these elements. Prior studies of TADs and their biological roles have revealed correlations between TAD changes and cellular differentiation. Here, we used Hi-C and RNA-seq data to correlate TCR-induced changes in TAD structure and gene expression in human CD4^+^ T cells.

**Results:**

We developed a pipeline, Differentially Expressed Gene Enrichment Finder (DEGEF), that identifies regions of differentially expressed gene enrichment. Using DEGEF, we found that TCR-regulated genes cluster non-uniformly across the genome and that these clusters preferentially localized in regions of TAD rearrangement. Interestingly, clusters of upregulated genes preferentially formed new Hi-C contacts compared to downregulated clusters, suggesting that TCR-activated CD4^+^ T cells may regulate genes by changing stimulatory contacts rather than inhibitory contacts.

**Conclusions:**

Our observations support a significant relationship between TAD rearrangements and changes in local gene expression. These findings indicate potentially important roles for TAD rearrangements in shaping their local regulatory environments and thus driving differential expression of nearby genes during CD4^+^ T cell activation. Moreover, they provide new insights into global mechanisms that regulate gene expression.

**Supplementary Information:**

The online version contains supplementary material available at 10.1186/s12864-023-09693-8.

## Background

Gene expression is known to be controlled by networks of interactions between transcription factors and DNA regulatory elements including enhancers and promoters [1–3]. While enhancers may be located kilobases or megabases away from the genes they regulate or even on different chromosomes, they are typically in reasonable genomic proximity and interact with their target genes through changes in nuclear architecture. However, how genome organization influences the function of transcriptional enhancers and how it drives spatiotemporal regulation of gene transcription remain unclear and are active areas of investigation [4].

Topologically associating domains (TADs) are self-interacting regions of the genome that influence the accessibility of local DNA regulatory elements including enhancers and promoters [5, 6]. TADs physically restrict regulatory activities involving enhancer-promoter interactions to specific, large regulatory domains and help to establish specific gene expression profiles [7]. Disruption of TAD boundaries can eliminate old enhancer-promoter contacts and form new ones, resulting in changes in local gene expression [8, 9]. Furthermore, disruption of TAD boundaries has been associated with many phenotypic outcomes and diseases, including cancer [10–12] and malformations in limb development [13, 14].

One process that naturally involves changes in TAD boundaries and structure is T cell receptor (TCR) activation of primary immune cells [15]. Upon TCR activation with anti-CD3 and anti-CD28 antibodies, CD4^+^ T cells undergo changes in gene expression and chromatin remodeling, resulting in differentially expressed genes (DEGs) and altered TADs [15–17]. However, how DEGs relate to changes in TADs in CD4^+^ T cells remains poorly understood. To date, some correlations between DEGs and TAD changes have been observed in similar processes. Previously, investigators found genome-wide changes in chromatin accessibility, TADs, and A/B compartments (the A compartment is associated with open active chromatin and the B compartment with closed inactive chromatin) during T cell commitment [18]. Moreover, a second group observed that enhancers and genes connected by loops had higher correlations between gene expression and H3K27 acetylation in human THP1 cells [19], while a third group used tagmentation-based Hi-C to map spatiotemporal dynamics of chromatin structure in hematopoietic stem and progenitor cells and myeloid differentiated cell populations and found that gene-body associating domains were structures of highly expressed genes [20].

Here, we examine the identities of DEGs longitudinally at 20 min, 1 h, 4 h, and 24 h after TCR activation of CD4^+^ T cells, their distribution throughout the genome, and their relationship to changes in genome organization via TAD rearrangement. To this end, we developed a new algorithm named Differentially Expressed Gene Enrichment Finder (DEGEF), which identifies regions with significant enrichment of DEGs. We used DEGEF to identify clusters of DEGs at different time points following TCR stimulation of CD4^+^ T cells. We then compared the genomic locations of these clusters to those of TAD rearrangements as determined by Hi-C sequencing of the same samples. Strikingly, multiple clusters of DEGs overlapped regions of TAD rearrangement. To our knowledge, this represents the first systematic analysis in CD4^+^ T cells of relationships between chromatin organization at the TAD level and gene expression changes in the context of genomic localization.

## Results

**Fig. 1:**
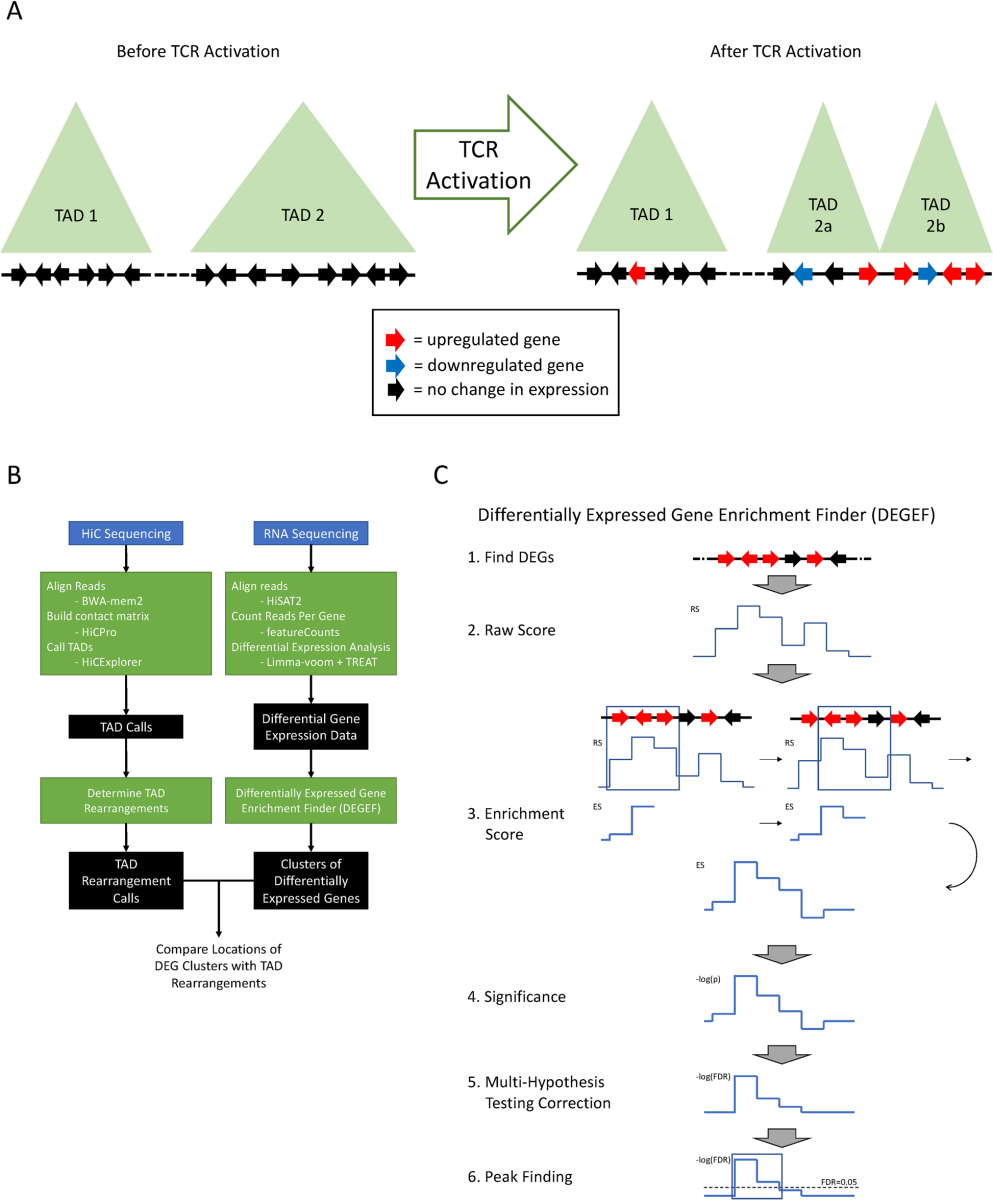
A computational approach exploring relationships between changes in TADs and gene expression. **A** Hypothesized relationship between genome compartmentalization and differential gene expression. Upon T-cell activation, TAD 1 does not change, whereas TAD 2 splits into two smaller TADs. Meanwhile, fewer changes in gene expression occur within TAD 1 than TAD 2 (see red and blue arrows). **B** Schematic of the analysis pipeline. Hi-C data from TCR-activated CD4^+^ T cells were processed using BWA-mem2, HiCPro, and HiCExplorer to identify TADs and TAD rearrangements during TCR activation. RNA-seq data from the same CD4^+^ T cells performed in duplicate were simultaneously processed using HiSAT2, featureCounts, and limma-voom with TREAT to identify DEGs during TCR activation. Then, DEGEF used the identified DEGs to identify genomic clusters of DEG enrichment. Finally, the locations of these TAD rearrangements and DEG clusters were compared to each other. **C** Schematic of DEGEF. First, limma-voom with TREAT identifies DEGs. Next, DEGEF computes a “raw score” (RS) of the degree of differential expression for each gene. It then sums the RSs in sliding, uniformly spaced genomic windows to compute an enrichment score (ES) for each locus. Finally, DEGEF computes *p*-values for each ES to determine significance, corrects for multiple hypothesis testing, and finally identifies peaks using an FDR threshold (e.g., FDR = 0.05)

Given the known relationship between DNA accessibility and gene expression, we hypothesized that CD4^+^ T cell activation results in changes in chromatin organization, which potentially then modulate changes in gene expression. Specifically, we conjectured that following T cell receptor activation of CD4^+^ T cells by stimulation with anti-CD3 and anti-CD28, genomic regions with significant changes in TAD structure would contain more DEGs (Fig. 1A), and genomic regions with a higher density of DEGs would be more likely to have undergone a TAD rearrangement than genomic regions with a lower density of DEGs.

To test this hypothesis, we developed a computational pipeline, DEGEF, to discover clusters of differential gene expression and TAD rearrangements from RNA-seq and Hi-C sequencing data, respectively, and then compare their overlap (Fig. 1B). We used this pipeline to analyze data from human CD4^+^ T cells before and after TCR activation [17]. (For a more detailed description of the dataset used, see Methods). In brief, after aligning the Hi-C sequencing data, the pipeline used HiCPro [21] to build and normalize Hi-C contact matrices, which were then passed to HiCExplorer [22, 23] to call TADs across the genome. We then compared TAD boundaries before versus after TCR activation to identify TAD rearrangements (see Methods for details on identifying TAD rearrangements). For RNA-seq data, the pipeline aligned the sequenced reads and then performed differential gene expression analysis using limma-voom with *t*-tests relative to a threshold (TREAT) [24–26] to identify DEGs and compute their fold changes (FCs) and statistical significances. Our pipeline passed these results to DEGEF (Fig. 1C) to identify regions enriched in upregulated or downregulated genes. In brief, DEGEF assigns each gene a “raw score” based on the level of upregulation or downregulation measured by limma-voom. A series of sliding genomic windows is used to sum the raw scores in each window to compute “enrichment scores” for loci throughout the genome, and the significance of each enrichment score is then computed. After correction for multiple hypothesis testing, series of contiguous windows exceeding a given False Discovery Rate (FDR) threshold were identified as clusters of DEG enrichment (see Methods for a comprehensive description and characterization of DEGEF). Thus, we identified clusters of upregulation and clusters of downregulation across the genome upon TCR activation of CD4^+^ T cells.

### Characterization of clusters of DEGs after TCR activation

We used published data for CD4^+^ T cells isolated from peripheral blood mononuclear cells from three healthy donors, where cells either were stimulated or not stimulated with anti-CD3 and anti-CD28 [17]. RNA-seq and Hi-C were performed prior to stimulation and at 20 min, 1 h, 4 h, and 24 h (single sample for 24 h; other time points were performed in duplicate) after stimulation. Overall, we observed similar changes in gene expression at these time points in CD4^+^ T cells following TCR activation. Specifically, the majority of DEGs at 4 h post-activation were also identified at 24 h post-activation, with general increases in FC and significance with time post-activation. (see Supplementary Text Sect.  9.1 and Supplementary Fig. S1 for a more comprehensive description of DEGs identified by RNA sequencing).

From these identified DEGs, we used DEGEF to identify clusters of upregulated and of downregulated genes in T cells 24 h after activation compared to unstimulated T cells. Using a window width size of 500 kb, a window step size of 20 kb, an FDR threshold of 0.05, and quantifying each gene’s raw score as the log_2_(FC) in expression, we identified 17 clusters of upregulated genes and 48 clusters of downregulated genes (Supplementary Tables 1 and 2) among 2505 upregulated genes and 2016 downregulated genes at 24 h post-activation. The average cluster of upregulated genes spanned 1.25 Mb and contained 7 upregulated genes. The average cluster of downregulated genes spanned 1.22 Mb and contained 5 downregulated genes. Additionally, when we ran DEGEF in “mixed” mode to identify clusters of DEGs regardless of direction (i.e., with either upregulation or downregulation), we observed strong concordance with the results of running DEGEF separately in upregulated and downregulated modes (see Supplementary Text Sect. 9.2 and Supplementary Table 3).

**Fig. 2:**
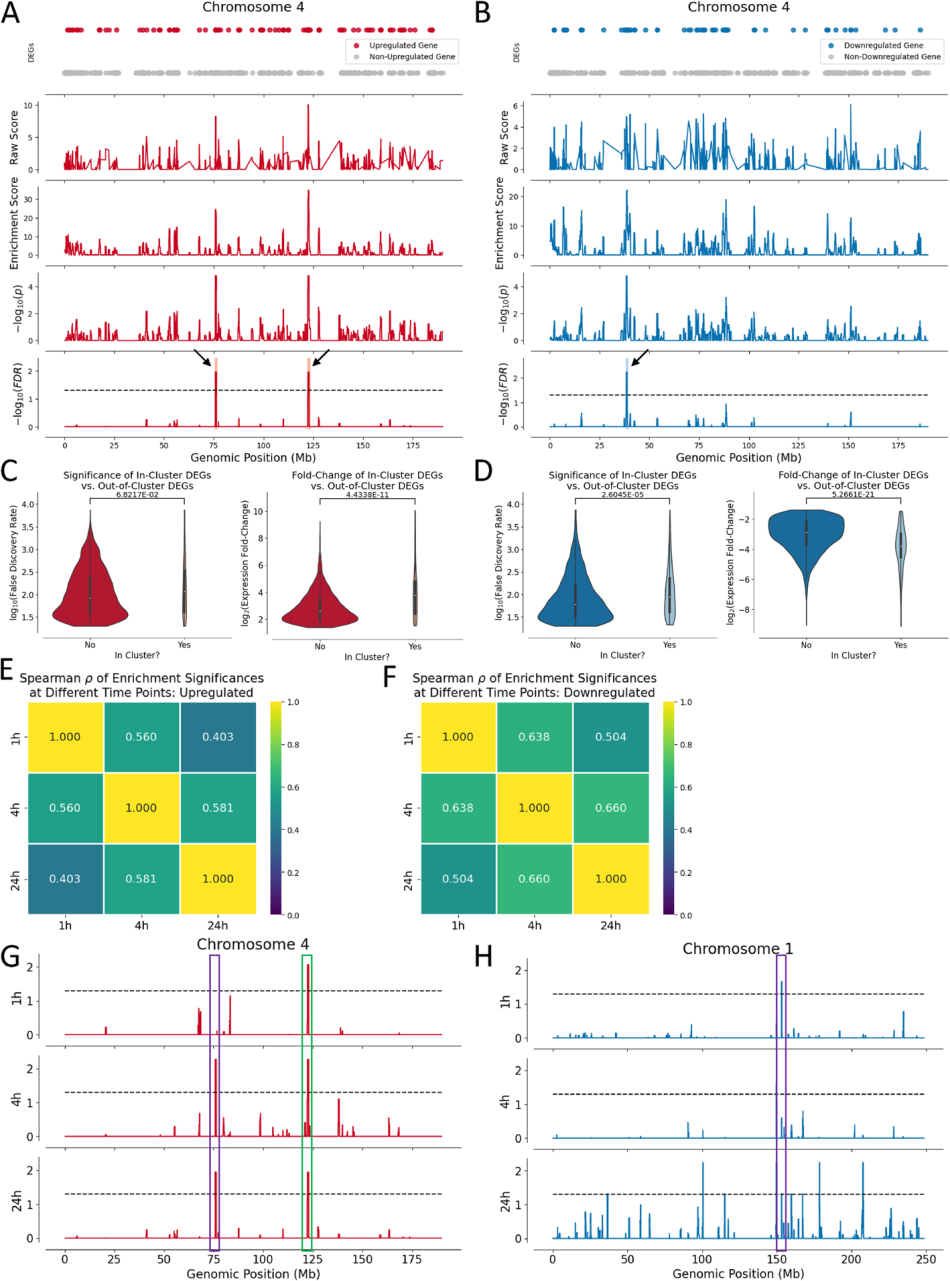
Identification and characterization of clusters of DEGs from RNA sequencing data. **A** Clusters of upregulated genes on chromosome 4. From top to bottom, we visualize: 1) transcription start sites of upregulated (red) or not upregulated (gray) genes, 2) raw scores of upregulated genes, 3) enrichment scores of genomic loci spanning this chromosome, 4) -log_10_(p) of the enrichment scores for each locus, and 5) -log_10_(FDR) for each locus, with clusters surpassing FDR < 0.05 (dashed line) indicated by black arrows and red shading. **B** Cluster of downregulated genes (blue dots) on chromosome 4. The identified cluster with FDR < 0.05 (dashed line, bottom plot) is marked with a black arrow and blue shading. **C** Violin plots showcasing changes in FC and significance between upregulated genes within upregulated clusters versus those outside these clusters. **D** Violin plots showcasing changes in FC and significance between downregulated genes within downregulated clusters versus those outside these clusters. **E** Correlation matrices depicting Spearman’s coefficients between significances (*p*-values) of enrichment of upregulation across the genome at 1, 4, and 24 h after TCR activation. **F** Correlation matrices depicting Spearman’s coefficients between significances (*p*-values) of enrichment of downregulation across the genome at 1, 4, and 24 h after TCR activation. **G** Evolution of upregulated clusters on chromosome 4 at 1, 4, and 24 h after TCR stimulation. Each track plots -log(enrichment FDR) at a different time after TCR activation. The cluster at 122.12 to 123.6 Mb (green) is significant at all time points, whereas the cluster at 75.58 to 77 Mb (purple) is significant only at 4 and 24 h after activation. **H** Evolution of downregulated clusters on chromosome 1 at 1, 4, and 24 h after TCR stimulation. Each track plots -log(enrichment FDR) at a different time after TCR activation. The cluster at 152.9 to 153.1 Mb (purple) is significant only at 1 and 24 h after activation

As representative examples of DEGEF’s ability to search for clusters of upregulated genes, we visualized below two of the seventeen total clusters of upregulated genes identified across the genome surpassing the FDR = 0.05 significance threshold. These two clusters were on chromosome 4: one from 75.58 to 77 Mb and another from 122.12 to 123.6 Mb (FDR = 1.13e-2; Fig. 2A). Known immunomodulator genes in these clusters include *CXCL10*, *CXCL11*, *SEPTIN11*, *SDAD1*, and *USO1* in the first cluster and *IL2*, *IL21*, *FGF2*, *SPRY1*, *SPATA5*, and *ADAD1* in the second cluster (see arrows). Overall, at 24 h after TCR activation, 17 clusters enriched for upregulated genes were identified on 13 different chromosomes (Supplementary Table 1). We also visualized one representative cluster of downregulated genes on chromosome 4 from 38.18 to 39.5 Mb out of 48 total downregulated clusters across the genome 24 h after TCR activation (FDR = 5.71e-3; Fig. 2B, see arrow). Known immunomodulators within this cluster that were downregulated include *LINC01259*, *KLF3*, *TLR10*, and *TLR1*. Overall, at 24 h after TCR activation, 48 clusters enriched for downregulated genes were identified on 19 different chromosomes (Supplementary Table 2).

DEGs within clusters exhibited greater changes in gene expression than DEGs outside clusters after TCR activation. Upregulated genes within clusters were associated with slightly lower *p*-values (Mann–Whitney U (MWU) test, *p* = 0.0682) and much higher log_2_(FC) values (MWU test, *p* = 4.43e-11) than upregulated genes outside these clusters (Fig. 2C). Meanwhile, downregulated genes within clusters were associated with both lower *p*-values (MWU test, *p* = 2.60e-5) and lower log_2_(FC) values (MWU test, *p* = 5.27e-21) than downregulated genes outside these clusters (Fig. 2D). These observations likely reflect DEGEF’s use of either log_2_(FC) or significance to score genes when identifying clusters of DEGs.

To assess how DEGEF-identified clusters evolved with time following TCR activation, we examined the correlation between “enrichment significances” (i.e., *p*-values) at all genomic loci at 1, 4, and 24 h after TCR simulation. Moderate correlation was observed for both upregulation and downregulation. For upregulation, higher correlation coefficients were observed between 1 and 4 h (Spearman’s rank correlation coefficient *ρ* = 0.560) and between 4 and 24 h (Spearman’s *ρ* = 0.581) than between 1 and 24 h after TCR stimulation (Spearman’s *ρ* = 0.403; Fig. 2E). Similarly, for downregulation, correlation coefficients between 1 and 4 h (Spearman’s *ρ* = 0.638) and 4 and 24 h (Spearman’s *ρ* = 0.660) were higher than observed between 1 and 24 h after TCR stimulation (Spearman’s *ρ* = 0.504; Fig. 2F). Based on the identities of the individual DEGs present at different timepoints after TCR activation, we believe that these observed differences in DEG clustering reflect real differences in gene expression profiles over the course of 24 h after TCR activation rather than noise inherent to our analysis.

Finally, we sought to determine how a subset of DEGEF-identified clusters of upregulated genes would evolve after TCR activation. We thus plotted the upregulated FDRs of genomic loci on chromosome 4 (Fig. 2G) and downregulated FDRs of loci on chromosome 1 (Fig. 2H) at 1, 4, and 24 h following TCR activation. Certain clusters were significant at 1 h and remained significant at 24 h after TCR activation (e.g., upregulation cluster on chromosome 4 from 122.12 to 123.6 Mb; highlighted in green), whereas other clusters either waxed (e.g., upregulation cluster on chromosome 4 from 75.58 to 77 Mb; highlighted in purple) or waned (e.g., downregulation cluster on chromosome 1 from 152.9 to 154.1 Mb; highlighted in purple) in significance. These dynamic changes in cluster significance and localization corresponded to observed significant changes in the gene expression profiles of the CD4^+^ T cells following TCR activation.

We also further evaluated DEGEF using an independent RNA-seq dataset from Th17 differentiated CD4^+^ T cells [27]. We observed both similarities with shared clusters and differences with distinctive clusters when we compared these results to the more neutrally TCR-activated CD4^+^ T cells described above (see Supplementary Text Sect. 9.3 and Supplementary Tables 4 and 5).

### Characterization of TAD rearrangements following TCR activation

We next used Hi-C data to examine TADs within unstimulated CD4^+^ T cells and how they changed after TCR activation. We used HiCPro to generate Hi-C contact matrices and HiCExplorer to call TADs. Past studies have demonstrated variability in median size and number of TADs called by different algorithms and from contact matrices binned at different resolutions [28]. We compared results using TADbit [29], TopDom [30], and Insulation Score [31] (see Supplementary Text Sect.  9.4 and Supplementary Fig. S2 for an overview of these comparisons). We observed slight differences in the TAD boundaries called by these different methods, but the TAD calls from HiCExplorer most faithfully matched previous reports of median TAD size and number in this dataset. Via visual inspection of HiCExplorer’s TAD calls, we noted these TADs correlated well with regions of high or low gene density and with principal components 1 and 2 from linear decomposition of the Hi-C contact matrix. (see Supplementary Text Sect.  9.5 and Supplementary Fig. S3 for an in-depth examination of a representative genomic region).

**Fig. 3:**
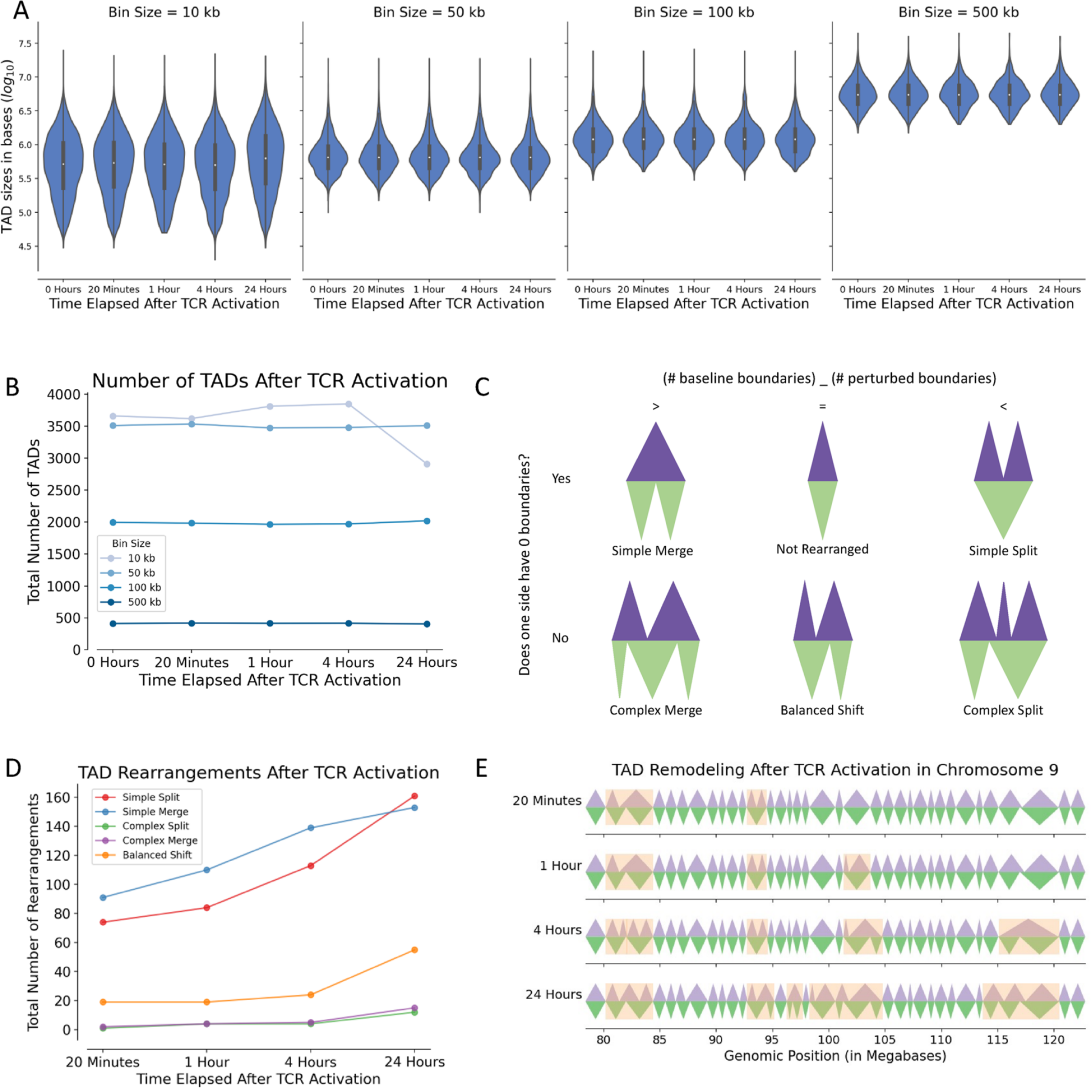
Identification and characterization of TAD rearrangements from Hi-C sequencing data. **A** Violin plots summarizing TAD sizes over time after TCR activation and using different bin sizes during Hi-C processing. The average TAD size did not substantially change following TCR stimulation; however, user-selected choice of bin size positively correlated with the resulting TAD sizes. **B** Total number of TADs present over time after TCR activation, stratified by choice of bin size used for Hi-C processing. The total number of TADs remained constant over time post-activation using bin sizes of 50–500 kb. However, increased variability in TAD number was observed with the lowest bin size (10 kb), perhaps due to increased stochasticity of the Hi-C contact matrix at higher resolution, as fewer reads map to each matrix bin. **C** Possible categories of TAD rearrangements: “simple merge,” “not rearranged”, “simple split”, “complex merge,” “balanced shift”, or “complex split,”. Classification depended on the number of TAD boundaries within the rearrangement before (green) versus after (purple) TCR activation. **D** Number of each type of TAD rearrangement observed over time after TCR activation. All types monotonically increased with time post-activation. Simple merges and simple splits were the most common types of rearrangements. **E** Visualization of TAD rearrangements (orange) in chromosome 9: ﻿78.4 Mb –122.8 Mb at different times after TCR activation, highlighting differences between pre-activation TADs (green) and post-activation TADs (purple). Rearranged regions tended to remain dynamic and continue to rearrange at later time points

Although many TAD boundaries shifted following TCR stimulation, median TAD size remained similar (Fig. 3A) and positively correlated with the choice of contact matrix resolution during processing (i.e., bin size) (Fig. 3A). The overall number of TADs also did not substantially change over time post-activation (Fig. 3B); however, at higher resolution (smaller bin sizes), the number of TADs varied greatly between 1, 4, and 24 h after TCR activation (Fig. 3B), likely due to increased stochasticity at smaller bin sizes. We selected a bin size of 100 kb for downstream analyses, as the resulting TADs were consistent with a prior report on the number and size of TADs in unstimulated and stimulated CD4^+^ T cells [15]. At this resolution, the median TAD size was 1.2 Mb and did not change significantly with TCR activation, and the total number of TADs increased modestly from 1991 pre-activation to 2016 at 24 h post-activation (Fig. 3B). Although the total numbers of TADs did not change significantly, we observed interesting changes in their boundaries. We classified these TAD rearrangements as “simple merge,” “simple split”, “complex merge,” “complex split,” or “balanced shift”, or “not rearranged” (Fig. 3C). Rearrangements were named “simple” if a single TAD completely spanned the rearrangement either before (bottom triangles, green) or after (top triangles, purple) TCR activation versus “complex” if more than one TAD always spanned the rearrangement. We named rearrangements “merges” if they contained more TADs before activation than after activation, whereas “splits” contained more TADs after activation. Finally, regions in which an equal number of TADs spanned the region before and after activation were named “balanced shifts.” The total number of all rearrangement types increased with time post-activation (Fig. 3D). Simple merges and simple splits were the most frequent TAD rearrangements, with simple merges being slightly more common than simple splits except at 24 h post-activation. Complex splits and complex merges were the least common TAD rearrangements and were observed only after 4 h post-activation. For illustrative purposes, we visualized TAD rearrangements within a region of the genome on chromosome 9 from 78.4 to 122.8 Mb (Fig. 3E) and observed that regions that undergo TAD rearrangement tend to remain dynamic, with TAD structures continuing to evolve through 24 h after TCR activation.

### Co-localization of DEG clusters with TAD rearrangements

**Fig. 4:**
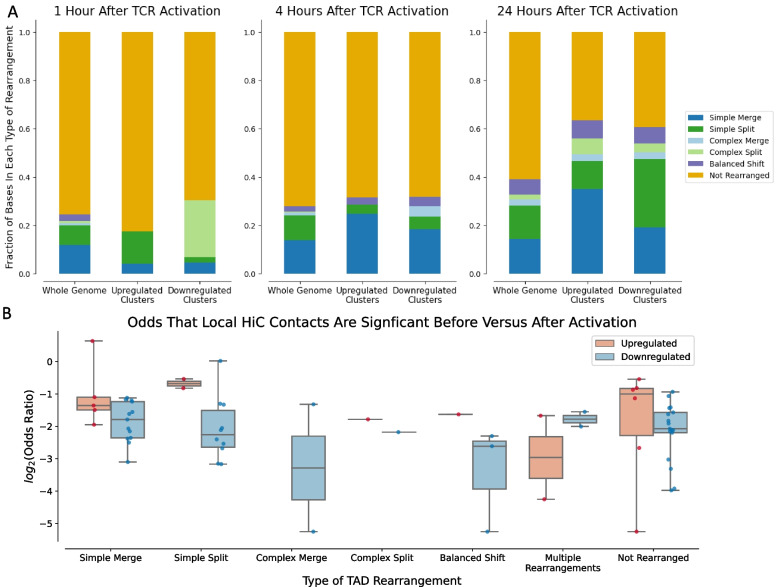
Global associations between TAD rearrangements and DEG clusters. **A** Stacked barplots demonstrating the percentage of bases within the entire genome, within upregulated clusters only, and within downregulated clusters only that fall within each type of TAD rearrangement at 1, 4, and 24 h after TCR activation. By 24 h, both upregulated and downregulated clusters significantly overlapped with TAD rearrangements. Simple merges are enriched in upregulated peaks, while simple splits are enriched in downregulated peaks. **B** Boxplot of odds ratios comparing the odds that any Hi-C loop contacting a DEGEF-identified cluster is significant after TCR activation versus the odds that it is significant before TCR activation. Aggregated across all types of rearrangements, upregulated clusters had significantly higher odds ratios than downregulated clusters (MWU *p* = 1.046e-2)

We next assessed the extent of overlap between DEGEF-identified clusters of upregulated or downregulated genes and TAD rearrangements. Across the genome, an increasing percentage of loci underwent TAD rearrangement from 1 to 24 h post-activation (﻿24.6%, 28.0%, 39.1% of loci at 1, 4, and 24 h respectively); however, when we considered only regions within DEGEF-identified clusters of upregulated or downregulated genes, we noted that most of the loci within these clusters had undergone TAD rearrangement by 24 h (Fig. 4A). At 1 and 4 h after TCR activation, the fraction of DEGEF-identified upregulated and downregulated clusters that had undergone TAD rearrangement was similar to the fraction of the genome that had undergone TAD rearrangement, indicating no significant correlation between TAD rearrangement and enrichment of differential gene expression at these time points. However, at 24 h post-activation, whereas only 39.1% of the whole genome was encompassed by TAD rearrangements, 63.5% of regions within upregulation clusters and 60.7% of regions within downregulation clusters overlapped TAD rearrangements, with more than twofold-enrichment of “simple merges” within upregulated clusters (14.2% of whole genome versus 35.1% of upregulation clusters) and of “simple splits” within downregulated clusters (13.9% of whole genome versus 28.5% of downregulation clusters).

We next used FitHiChIP [32] to assess the significance of all Hi-C contacts and computed the odds ratios that the Hi-C contacts in DEGEF-identified regions were significant before versus after TCR activation. Except in clusters overlapping multiple rearrangements, the odds ratios corresponding to upregulated clusters were generally higher than those corresponding to downregulated clusters (Fig. 4B), suggesting a tendency for new, statistically significant contacts to form in upregulated clusters compared to downregulated clusters. This trend persisted (median log_2_(FC) = 1.35 for simple merges and 2.99 for simple splits) after stratifying by type of TAD rearrangement, despite not achieving statistical significance (MWU *p* = 0.0945 for simple merges and *p* = 0.1212 for simple splits). Thus, in TCR-activated CD4^+^ T cells, gene upregulation may be driven mostly by formation of new stimulatory contacts rather than dissolution of existing inhibitory contacts, whereas downregulation may be driven largely through elimination of stimulatory contacts rather than formation of new inhibitory contacts.

Additionally, we assessed changes in chromatin accessibility as measured by ATAC-seq within DEGEF-identified clusters of DEGs. We defined ATAC-seq peaks called by MACS2 [33] that surpassed a threshold of FDR < 0.05 as regions of open chromatin. We observed an increase in open chromatin regions within DEGEF-identified clusters of upregulation, from 582 peaks pre-activation to 625 peaks post-activation. Conversely, we also observed a decrease in open chromatin regions in DEGEF-identified clusters of downregulation, from 1443 peaks pre-activation to 1390 peaks post-activation. Thus, upon TCR activation, increased chromatin accessibility likely leading to increased transcriptional activity was observed at clusters of upregulation. In contrast, decreased chromatin accessibility likely leading to decreased transcriptional activity was observed at clusters of downregulation.

**Fig. 5:**
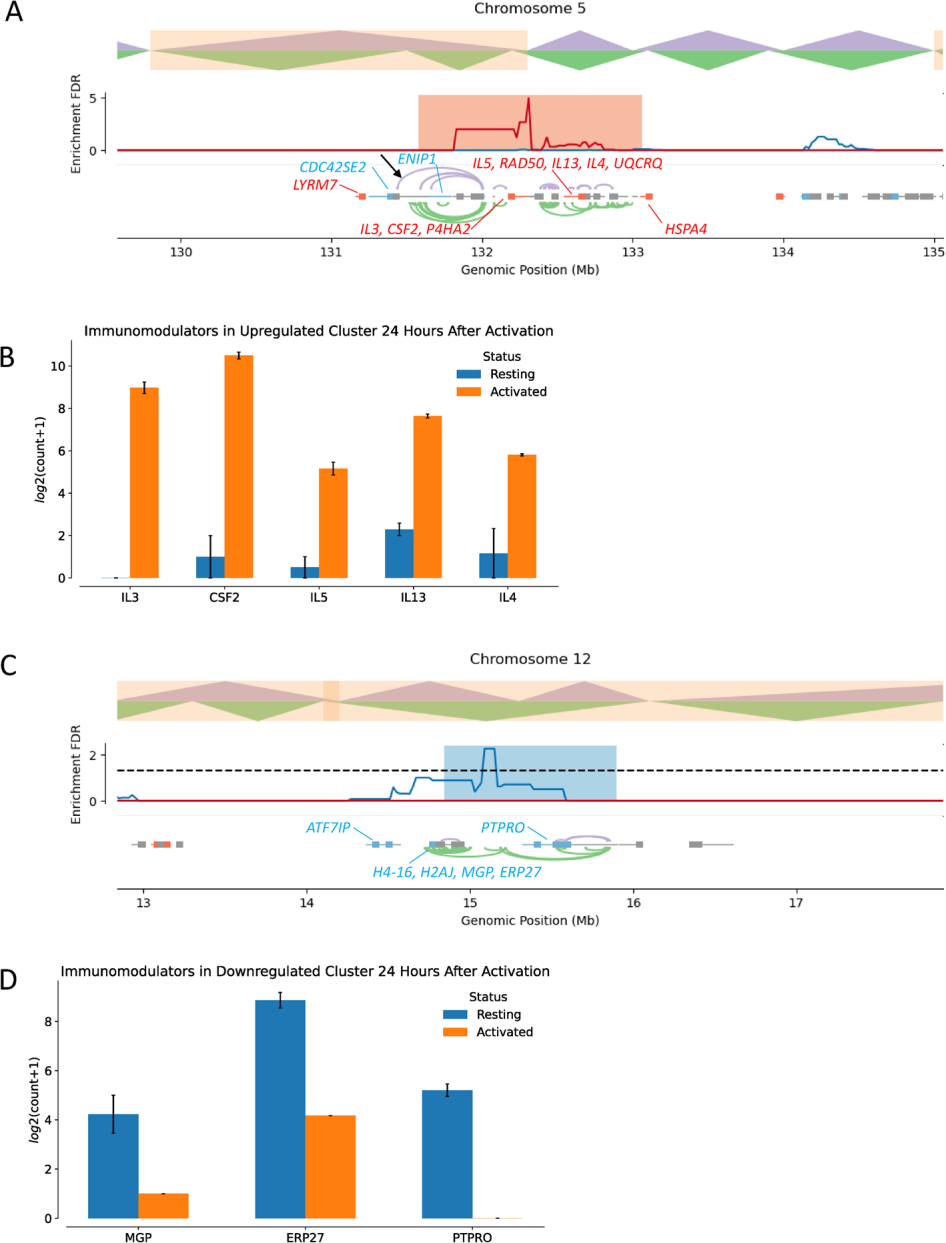
Specific DEGEF-identified clusters overlap with TAD rearrangements. **A** Example of a DEGEF-identified cluster of upregulation on chromosome 5 from 131.6 Mb to 133.0 Mb that co-localizes with a simple merge at 24 h after TCR activation. Significant Hi-C contacts as determined by FitHiChIP before (green) and after (purple) TCR activation are shown in the bottom track. The black arrow indicates the formation of a new significant Hi-C contact spanning two formerly separate TADs. Upregulated (red) and downregulated (blue) genes are also shown. **B** Upregulated immunomodulators within the DEGEF-identified cluster shown in **A**. *IL3*, *CSF2*, *IL5*, *IL13*, and *IL4* are all upregulated at 24 h after activation (orange) compared to before activation (blue). **C** Example of a DEGEF-identified cluster of downregulation on chromosome 12 from 14.84 Mb to 15.9 Mb that co-localizes with a simple split at 24 h after TCR activation. Significant Hi-C contacts as determined by FitHiChIP before (green) and after (purple) TCR activation are shown in the bottom track. Upregulated (red) and downregulated (blue) genes in this region are also shown. **D **Downregulated immunomodulators within the DEGEF-identified cluster shown in **C**. *MGP*, *ERP27*, and *PTPRO* are all downregulated at 24 h after activation (orange) compared to before activation (blue)

Finally, we examined representative examples of overlap between DEGEF-identified clusters of upregulated and downregulated genes and TAD rearrangements. One stretch on chromosome 5 (131.58 to 133.06 Mb) contained a cluster of upregulated genes that co-localized with a “simple merge” 24 h after TCR activation (Fig. 5A). Differentially expressed immunomodulator genes within this cluster included *IL3*, *CSF2*, *IL5*, *IL13*, and *IL4* (Fig. 5B). A new significant Hi-C loop from the 5 kb bin starting at 131.435 Mb to the 5 kb bin starting at 132 Mb (black arrow in Fig. 5A) formed within this cluster after TCR activation. We hypothesized that this new loop formed during TAD merging and may bridge regulatory elements present in the first pre-activation TAD (129.8 to 131.5 Mb) to DEGs present in the second, adjacent TAD (131.5 to 132.2 Mb), thus driving increased gene expression. Another stretch on chromosome 12 (14.84 to 15.9 Mb) contained a cluster of downregulated genes that co-localized with a “simple split” 24 h post-activation (Fig. 5C). DEGs within this cluster included *MGP*, *ERP27*, and *PTPRO* (Fig. 5D). Conceivably, regulatory elements in each of the two newly formed TADs (14.2 to 15.3 Mb and 15.3 to 16.1 Mb) no longer had access to the genes present in the other TAD, as evidenced by decreased Hi-C looping after TCR activation. The loss of such interactions possibly contributed to downregulation of genes spanning the original TAD from 14.1 to 16.1 Mb that existed prior to TCR activation.

## Discussion

In this manuscript, we used a new pipeline, DEGEF, to identify clusters of DEGs upon TCR activation in CD4^+^ T cells. In earlier work, an existing algorithm, DERFinder, had been developed to discover novel, unannotated small genomic regions of differential expression, such as previously unknown exons, at single-base resolution [34, 35], and its usage has been limited to this particular context. In contrast, our pipeline DEGEF is a first-of-its-kind algorithm that identifies clusters of upregulated and downregulated genes spanning relatively large genomic regions; to our knowledge, such features are not available in any other tool. Moreover, we found that these regions of differentially expressed genes correlate well with regions undergoing large-scale changes in chromatin structure via TAD rearrangement in biologically relevant contexts. Thus, our tool offers previously unavailable insight into genomic regions of higher than average differential gene expression and chromatin-related structure–function relationships associated with these regions.

Interestingly, increased numbers of downregulated genes with time after TCR activation led to a corresponding increase in the number of clusters of downregulated genes, whereas increased numbers of upregulated genes led to the identification of approximately the same number of clusters of upregulated genes. Thus, genomic processes with a strong localization component, such as intra-TAD DNA looping and short-range enhancer-enhancer or enhancer-promoter effects, might play a stronger role in driving upregulation than downregulation following TCR stimulation of CD4^+^ T cells.

Prior studies of Hi-C and RNA-seq data from primary immune cells provide limited analyses of the changes in TAD organization and gene expression in CD4^+^ T cells upon TCR activation [15, 17]. While these studies noted trends in the number of TADs pre-activation that overlap with TADs post-activation and changes in median TAD size upon TCR activation, individual changes in TAD structure were not correlated with clusters of DEGs. Here, we correlated the observed gene expression changes with local rearrangements in TAD structure. Specifically, upon TCR activation, upregulated genes were more likely to be in regions where two TADs merged into a single TAD, whereas downregulated genes were more likely to be in regions where a single TAD split into two separate TADs. This suggests that TCR-induced gene upregulation might result from increased access to cis-regulatory elements such as enhancers or super-enhancers in merged TADs, whereas TCR-induced gene downregulation might result from decreased access to these cis-regulatory elements. Finally, we observed that the Th2 cytokine locus on chromosome 5 from 131 to 134 Mb remains a cluster of upregulation and is associated with local changes in chromatin structure during T cell activation, as has been observed in previous studies [36].

## Conclusions

In summary, we developed and used a novel algorithm DEGEF to identify clusters of upregulated and downregulated genes in TCR-stimulated CD4^+^ T cells and demonstrated that they co-localize with regions of TAD rearrangement. At 24 h after TCR activation, clusters of upregulation preferentially localized where two separate TADs had merged into a single TAD (simple merge), whereas clusters of downregulation preferentially localized where a single TAD had split into two separate TADs (simple split). We identified a subset of TAD rearrangements associated with significant changes in gene expression and that may have functional significance as potential modulators of immune cell gene expression. It is conceivable that agents that modulate TADs and their boundaries may comprise a novel class of therapeutics. Specifically, given the technology to disrupt TADs in any cell type, it may be possible to activate the immune system when inappropriately suppressed or suppress it when inappropriately activated. If developed, such TAD-modifying agents could have potential efficacy for many diseases, including cancer, where modulation and correction of dysregulated transcription is desirable.

## Methods

### Datasets used

To identify clusters of differentially expressed genes in CD4^+^ T cells following TCR stimulation, we used a published dataset in which CD4^+^ T cells isolated from peripheral blood mononuclear cells (PBMCs) from three healthy donors were stimulated with anti-CD3 and anti-CD28 [17]. RNA and Hi-C sequencing were performed before stimulation and at 20 min and 1, 4, and 24 h (single sample for 24 h; other time points were performed in duplicate) after stimulation. We identified DEGs and clusters of differential gene expression by using limma-voom and DEGEF, respectively, to compare gene expression at the four time points after TCR activation versus baseline gene expression prior to TCR activation. We used localization data for known genomic regions (i.e., transcription start sites) and RNA-seq data to define broad regions of the genome that contain more DEGs than are expected by chance. We used HiCPro to align, filter, and normalize the Hi-C sequencing data and then used HiCExplorer’s hicFindTADs function to identify TADs at each time point.

### Identifying TAD rearrangements

TAD rearrangements were defined as regions in which TAD structure varied between two different cellular treatment conditions, as assessed by Hi-C experiments conducted at various time points after TCR stimulation. To identify TADs, we used HiCPro to align, filter, and normalize Hi-C sequencing data and then used HiCExplorer’s hicFindTADs function. To identify TAD rearrangements, TAD calls were compared between different conditions (e.g., T cells 24 h after TCR stimulation versus unstimulated T cells). TAD boundaries were considered discordant if no corresponding boundary was found in the complementary TAD set within a 1-bin-width radius (e.g., within 100 kb for TADs called using a 100 kb contact matrix). All genomic regions between concordant TAD boundaries were identified as potential TAD rearrangements. TAD rearrangements spanning centromeres denoted in the UCSC human genome annotation (assembly GRCh38, hg38) were removed from further consideration, as the repetitive nature of these regions decreased the reproducibility of TAD calls in these regions.

Finally, depending on the number of TADs present within each potential rearrangement before and after cellular perturbation (i.e., TCR activation), five classes of rearrangements were defined as follows:

1. Simple merges: multiple TADs merge into one TAD
2. Simple splits: one TAD splits into multiple TADs
3. Complex merges: multiple TADs merge into two or more TAD(s), with the total number of TADs in the region decreasing
4. Complex splits: two or more TADs split into more TADs, with the total number of TADs in the region increasing
5. Balanced shifts: two or more TADs rearrange boundaries, resulting in the same number of TADs

Besides these rearrangements, TADs may also undergo no change (“Not rearranged”).

### DEGEF methodology

DEGEF is a computational pipeline that identifies clusters of high DEG enrichment across the genome. RNA sequencing (RNA-seq) data are aligned, processed, and assessed for differential gene expression using limma-voom with *t*-tests relative to a Threshold (TREAT), to assess differential gene expression exceeding a given fold-change (FC) threshold [24–26, 37]. The resulting table of genes, their computed FCs, *p*-values, and false discovery rates (FDRs) outputted by limma-voom is then passed to DEGEF to identify regions enriched in DEGs using the steps outlined below. DEGEF can be run in “upregulated,” “downregulated,” or “mixed” modes, depending on whether the user wishes to search for regions of significant upregulation, downregulation, or both.

DEGEF first computes a raw score (RS) for each gene using one of three methods: “count,” “significance,” or “foldchange.” Using “count” (default mode), DEGEF assigns a gene a value of 1 if it is upregulated (in “upregulated” mode) or downregulated (in “downregulated” mode) and 0 otherwise. Genes are classified as upregulated, downregulated, or not differentially regulated according to limma-voom results and user-inputted threshold parameters for adjusted *p*-value and FC. DEGEF uses an adjusted *p*-value threshold of 0.05 and a FC threshold of 2.0 as default parameters (i.e., a gene must have both an adjusted *p*-value ≤ 0.05 and FC ≥ 2 if upregulated or FC ≤ 0.5 if downregulated). In “mixed” mode, genes are assigned a value of 1 if they are either upregulated or downregulated and 0 otherwise. Mathematically, we represent these functions as:

1 $$R{S}_{count}\left(g\right)= \left\{\begin{array}{cc}1& if\,g\,is\,upregulated\\ 0& otherwise\end{array}\right.$$

2 $$R{S}_{count}\left(g\right)= \left\{\begin{array}{cc}1& if\,g\,is\,downregulated\\ 0& otherwise\end{array}\right.$$

3 $$R{S}_{count}\left(g\right)= \left\{\begin{array}{cc}1& if\,g\,is\,upregulated\\ 1& if\,g\,is\,downregulated\\ 0& otherwise\end{array}\right.$$

Using the “significance” method of raw scoring, genes are scored using the negative common logarithm of their adjusted *p*-values by limma-voom if they are upregulated (in “upregulated” mode) or downregulated (in “downregulated” mode) and 0 otherwise. In “mixed” mode, genes are scored using ${-\mathrm{log}}_{10}(\mathrm{FDR})$ if downregulated and ${-\mathrm{log}}_{10}(\mathrm{FDR})$ if upregulated. Mathematically, we represent these functions as:

4 $$R{S}_{significance}\left(g\right)= \left\{\begin{array}{cc}-{\mathrm{log}}_{10}(FDR\left(g\right))& if\,g\,is\,upregulated\\ 0& otherwise\end{array}\right.$$

5 $$R{S}_{significance}\left(g\right)= \left\{\begin{array}{cc}-{\mathrm{log}}_{10}(FDR\left(g\right))& if\,g\,is\,downregulated\\ 0& otherwise\end{array}\right.$$

6 $$R{S}_{significance}\left(g\right)= \left\{\begin{array}{cc}-{\mathrm{log}}_{10}(FDR\left(g\right))& if\,g\,is\,upregulated\\ -{\mathrm{log}}_{10}(FDR\left(g\right))& if\,g\,is\,downregulated\\ 0& otherwise\end{array}\right.$$

Using the “foldchange” method of raw scoring, genes are scored using ${\mathrm{log}}_{2}\left(\mathrm{FC}\right)$ if they are upregulated (in “upregulated” mode) and 0 otherwise or ${-\mathrm{log}}_{2}(\mathrm{FC})$ if downregulated (in “downregulated” mode) and 0 otherwise. In “mixed” mode, genes are scored as ${\mathrm{log}}_{2}(\mathrm{FC})$ if upregulated or -log_2_(FC) if downregulated and 0 otherwise. Mathematically, we represent these functions as:

7 $$R{S}_{foldchange}\left(g\right)= \left\{\begin{array}{cc}{\mathrm{log}}_{2}(FC\left(g\right))& if\,g\,is\,upregulated\\ 0& otherwise\end{array}\right.$$

8 $$R{S}_{foldchange}\left(g\right)= \left\{\begin{array}{cc}-{\mathrm{log}}_{2}(FC\left(g\right))& if\,g\,is\,downregulated\\ 0& otherwise\end{array}\right.$$

9 $$R{S}_{foldchange}\left(g\right)= \left\{\begin{array}{cc}{\mathrm{log}}_{2}(FC\left(g\right))& if\,g\,is\,upregulated\\ {-\mathrm{log}}_{2}(FC\left(g\right))& if\,g\,is\,downregulated\\ 0& otherwise\end{array}\right.$$

Next, we subdivide each chromosome arm into a series of equally spaced, equal-width genomic bins (or sliding windows). The width of and step-size between these windows are inputted by the user as two run-time parameters, with DEGEF using a default window width of 500 kb and a default step-size of 20 kb. (See Supplementary Text Sect. 9.6 and Supplementary Fig. S4 for an overview of how varying window size and raw scoring methodology affects DEGEF’s outputs). DEGEF computes an enrichment score (ES) for each window as the sum of the raw scores for all genes in that window:

10 $$ES\left(W\right)= {\sum }_{g\in W}RawScore(g)$$

DEGEF then assesses the statistical significance of each computed ES. Under the “count” method of raw scoring, each gene’s probability of upregulation or downregulation is modeled as an independent Bernoulli trial, with success probability estimated by the observed frequency of upregulation/downregulation of all genes across the genome. Thus, the null distribution for the sum of multiple such independent and identically distributed variables is the binomial distribution B(*n*, *p*), where *n* is the number of genes in the genomic window and *p* is the empiric probability of upregulation/downregulation of any gene in the genome. The *p*-value is computed as the survival function of this distribution’s normal approximation:

11 $$p\left(W\right)= {\int }_{ES(W)}^{\infty }\mathrm{Gauss}(np, np\left(1-p\right))dx$$

Under the “significance” and “fold change” methods of raw scoring, the null distribution is constructed empirically using bootstrapping [38] with replacement (i.e., randomly sampling and summing collections of *n* raw scores, where *n* is the number of genes in the present genomic window), if *n* was less than a user-specified threshold *t* (default *t* = 30). The total number of bootstraps *x* used to build each null distribution is defined by the user (default *x* = 6400). For computational convenience when assessing windows with *n* ≥ *t*, we instead use the Central Limit Theorem (CLT) to approximate the distribution of enrichment scores from the mean (μ) and standard deviation (σ) of the observed raw scores across the genome (see Supplementary Text Sect. 9.7 and Supplementary Fig. S5 for further details including characterization of the accuracy of this approximation). The *p*-value is then computed as the survival function of either the empirically constructed, bootstrapped null distribution (if *n* < *t*) or the CLT-approximated gaussian distribution (if *n* ≥ *t*):

12 $$p(W)= \left\{\begin{array}{cc}{\int }_{ES(W)}^{\infty }\mathrm{Boot}(ES\left(W, {n}_{gene}=n\right))& if n<t\\ {\int }_{ES(W)}^{\infty }\mathrm{Gauss}(\mu , \sigma )dx& if n\ge t\end{array}\right.$$

Correction for multiple hypothesis testing is performed using the Benjamini–Hochberg procedure to decrease the FDR by controlling for the fact that small *p*-values sometimes occur by chance. Finally, DEGEF identifies all contiguous genomic windows exceeding a user-specified FDR threshold (default FDR = 0.05). Each set of contiguous windows that fall beneath this threshold defines a distinct DEG enrichment cluster.

Of note, DEGEF’s outputs are dependent upon the choice of multiple user-defined parameters (e.g., window size, step size, scoring metric, etc.), which allows for versatility in cluster-calling. For example, using larger window sizes (e.g., 2.5 Mb) increases statistical power, as more genes are present in each window; however, using larger window sizes reduces the resolution of each cluster, as each cluster cannot be narrower than the width of a single window. Conversely, using smaller window sizes (e.g., 100 kb) increases resolution but at the cost of statistical power to detect enrichment due to fewer genes being within each window. Moreover, many of the clusters identified by DEGEF in our analyses remained conserved even when using different run-time parameters. It is possible that DEGEF’s run-time parameters will require adjustment depending on the number and distribution of DEGs to optimize the algorithm’s performance. We have retained the user-defined nature of these parameters to allow for heuristic selection based on the nature of the dataset being analyzed.

### Supplementary Information

**Additional file 1:** **Supplementary Figure S1.** Differential Gene Expression Results. **Supplementary Figure S2.** Measuring robustness of TAD calling using alternative TAD-calling algorithms. **Supplementary Figure S3.** Hi-C Contact Matrices. **Supplementary Figure S4.** DEGEF Robustness to Window Size Parameter. **Supplementary Figure S5.** Central Limit Theorem (CLT) can be used in DEGEF Modeling.

**Additional file 2:** **Supplementary Table 1.** Clusters of upregulation observed 24 hours after TCR activation in CD4+ T cells. Each column represents one cluster. The first row in each column reports the cluster’s genomic coordinates. The second row reports the lowest FDR within the cluster. Each of the following rows reports a gene within the cluster, with differentially expressed genes starred.

**Additional file 3:** **Supplementary Table 2.** Clusters of downregulation observed 24 hours after TCR activation in CD4+ T cells. Each column represents one cluster. The first row in each column reports the cluster’s genomic coordinates. The second row reports the lowest FDR within the cluster. Each of the following rows reports a gene within the cluster, with differentially expressed genes starred.

**Additional file 4:** **Supplementary Table 3.** Clusters of mixed differential gene expression observed 24 hours after TCR activation in CD4+ T cells. Each column represents one cluster. The first row in each column reports the cluster’s genomic coordinates. The second row reports the lowest FDR within the cluster. Each of the following rows reports a gene within the cluster.

**Additional file 5:** **Supplementary Table 4.** Clusters of upregulation 24 hours after TCR activation in Th17 polarizing conditions in CD4+ T cells. Each column represents one cluster. The first row in each column reports the cluster’s genomic coordinates. The second row reports the lowest FDR within the cluster. Each of the following rows reports a gene within the cluster, with differentially expressed genes starred.

**Additional file 6:** **Supplementary Table 5.** Clusters of downregulation 24 hours after TCR activation in Th17 polarizing conditions in CD4+ T cells. Each column represents one cluster. The first row in each column reports the cluster’s genomic coordinates. The second row reports the lowest FDR within the cluster. Each of the following rows reports a gene within the cluster, with differentially expressed genes starred.

## Data Availability

The datasets analyzed during the current study were generated as part of an existing study. These data are available in the National Center for Biotechnology Information’s Gene Expression Omnibus and are accessible through GEO Series accession number GSE138767 (https://www.ncbi.nlm.nih.gov/geo/query/acc.cgi?acc=GSE138767). Code used to generate results central to this article’s conclusions may be accessed as detailed below: Project name: Differentially Expressed Gene Enrichment Finder (DEGEF). Project home page: https://github.com/gaog94/DEGEF/ Archived version: https://zenodo.org/badge/latestdoi/600197447 Operating system(s): Platform independent. Programming language: Python3. Other requirements: numpy v1.21, pandas v1.4.4, statsmodels v0.13.2, scipy v1.9.1, matplotlib v3.5.2 License: MIT. Any restrictions to use by non-academics: None.

## Abbreviations

DEG: Differentially Expressed Gene
DEGEF: Differentially Expressed Gene Enrichment Finder
FC: Fold Change
FDR: False Discovery Rate
MWU: Mann-Whitney U test
TAD: Topologically Associating Domain
TCR: T-cell Receptor
TREAT: *t*-Tests Relative to a Threshold
