# Supplementary material for: Co-localization of clusters of TCR-regulated genes with TAD rearrangements
Source: BMC Genomics. 2023 Oct 28;24:650. doi: 10.1186/s12864-023-09693-8 (PMC10613383; doi:10.1186/s12864-023-09693-8)
Supplement: Supplementary file 1 — Additional file 1: Supplementary Figure S1. Differential Gene Expression Results. Supplementary Figure S2. Measuring robustness of TAD calling using alternative TAD-calling algorithms. Supplementary Figure S3. Hi-C Contact Matrices. Supplementary Figure S4. DEGEF Robustness to Window Size Parameter. Supplementary Figure S5. Central Limit Theorem (CLT) can be used in DEGEF Modeling. [file 12864_2023_9693_MOESM1_ESM.pdf]

9 Supplementary Text

9.1 DEGs identified from RNA sequencing data

We identified DEGs from the RNA-seq data at the post-activation time points compared to pre-activation with FC ≥ 2 and FDR ≤ 0.05 (**Supplementary Figure S1A;** data are shown only at 1, 4, and 24 hours, as at 20 minutes no genes satisfied our thresholds; the top 10 most significant genes at each time point are indicated in blue). Overall, the magnitude of changes in gene expression increased with time post-activation. To assess whether the same genes were differentially expressed throughout TCR activation, we examined the significance of change at 4 versus 24 hours post-activation (**Supplementary Figure S1B**). We observed distinct sets of genes that were significant at one time point but not at the other and a mild, positive correlation (Spearman's coefficient ρ=0.3773) between gene significances at both time points. Of the 855 genes differentially expressed at 4 hours, the majority (627, 73.3%) were also differentially expressed at 24 hours post-activation. Of these, only two genes (OASL and NR4A2) changed their directionality of expression and were upregulated at 4 hours but downregulated at 24 hours. Consistent with the general increase in DEGs over time, 257 genes were differentially expressed only at 4 hours while 3894 genes were differentially expressed only at 24 hours (**Supplementary Figure S1C**). Thus, we observed similar changes in gene expression at different time points in $CD4^+$ T cells following TCR activation, with a greater number and significance of differential gene expression at later time points.

Supplementary Figure S1

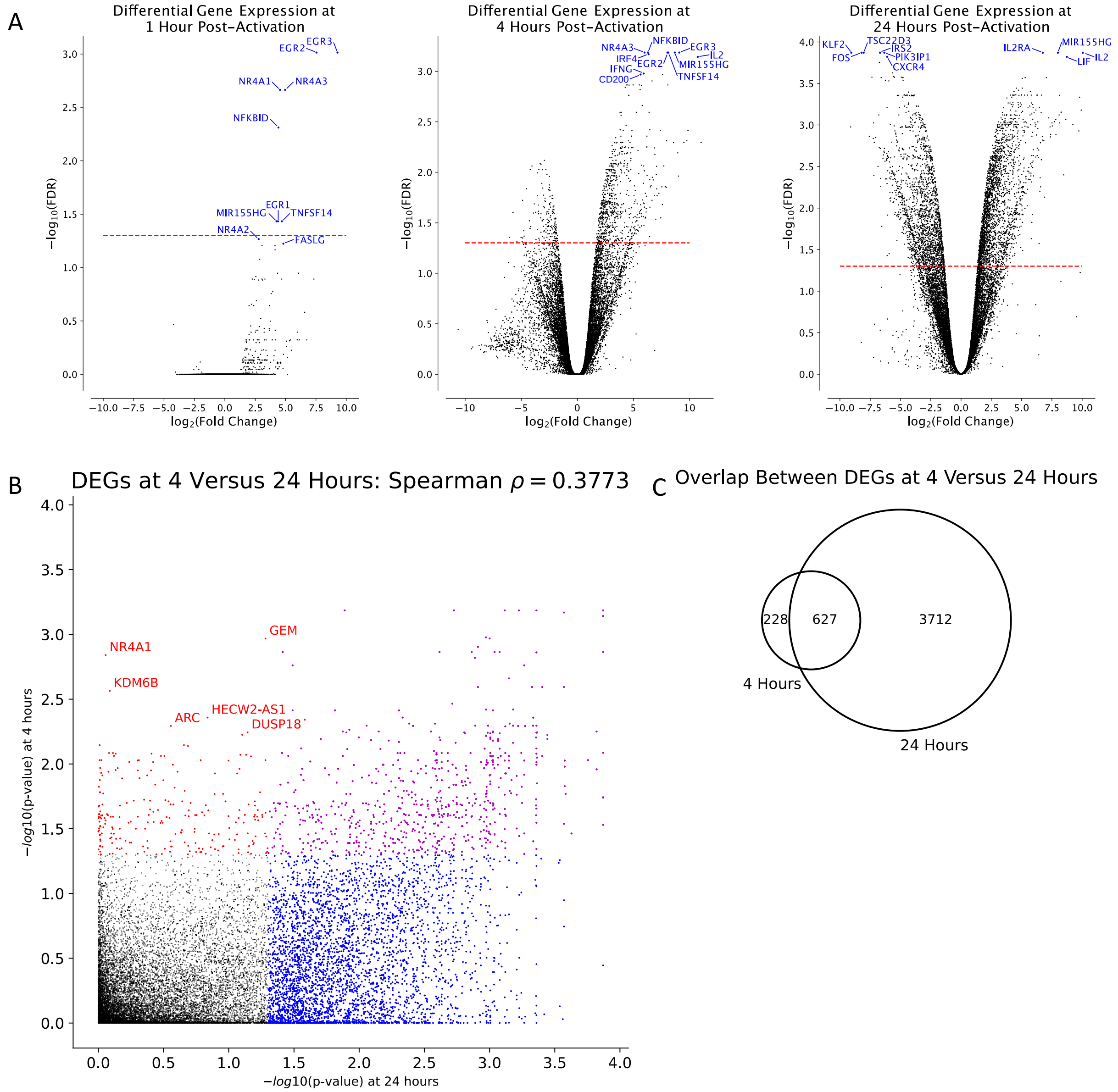

Supplementary Figure S1 – Differential Gene Expression Results

A) Volcano plots of differential gene expression at 1, 4, and 24 hours post-TCR activation. The top 10 most significant genes at each time point are labeled in blue.

B) Adjusted p-value at 4 versus 24 hours post-activation. We observed a positive correlation (Spearman's ρ=0.3773) between the two sets of p-values and substantial overlap between DEGs at 4 and 24 hours (purple dots). There are subsets of DEGs present only at 4 hours (red dots) and only at 24 hours (blue dots).

C) Venn diagram showing overlap between significantly differentially expressed genes (FDR < 0.05) at 4 hours versus 24 hours post-activation.

9.2 "Mixed" Mode DEGEF Analysis of $CD4^+$ T Cell Activation

Running DEGEF in "mixed" mode, we identified 14 clusters of increased differential gene expression in either direction (**Supplementary Table 3**). Of these 14 clusters, 13 had already been identified previously in our analysis as either clusters of upregulation or clusters of downregulation. The one novel peak identified by this mode was a cluster from 41.4 Mb to 42.4 Mb on chromosome 1 containing *HIVEP3* (upregulated), *FOXJ3* (marginally downregulated), *ENSG00000230881* (upregulated) and *RIMKLA* (upregulated).

9.3 Validation of DEGEF Using a Related Dataset

To further evaluate the performance of DEGEF, we ran DEGEF on a second dataset from a different study that also activated $CD4^+$ T cells using anti-CD3 and CD28 antibodies. (Zhao, et al., 2014) However, this activation was performed in the presence of Th17 polarizing cytokines and antibodies, rather than under neutral conditions, as in our original dataset. Our analysis of this validation dataset identified 7 clusters of upregulation and 20 clusters of downregulation (**Supplementary Tables 4 & 5** respectively). Of these clusters, 2 upregulation clusters and 5 downregulation clusters were explicitly identified in both datasets, and many more clusters shared proximity within 1-5 megabases. Among clusters explicitly identified in both datasets were a peak of upregulation at Chr4: 121.8 – 123.4 Mb containing *IL2, IL21, FGF2, EXOSC9, CCNA2,* and *BBS7* and a peak of downregulation at Chr1: 206.8 – 208.4 Mb containing *CR1, CR2, CD34, IL24, CD55, FCMR*, and *PLXNA2*. Examples of new clusters not identified in the original dataset but identified in the validation dataset included peaks of upregulation at Chr2: 112.2 – 113.2 Mb containing *IL1A, IL11B, ZC3H8, TTL, CKAP2L*, and *PSD4* and at Chr12: 67.4 – 69 Mb containing I*FNG, IL26, IL22, RAP1B, NUP107, MDM2*, and *CPM*. We believe that these results reflect both similarities and differences in activating $CD4^+$ T

cells under neutral TCR stimulation conditions with anti-CD3/anti-CD28 versus Th17 polarizing conditions in our original versus the validation datasets, respectively. DEGEF was able to identify both clusters of common DEGs shared between the two pathways and several clusters unique to each pathway.

9.4 Comparison of TAD Calls From TAD-Callers Besides HiCExplorer

To assess the quality of the Hi-C data in producing robust TAD calls, we computed TAD boundaries using several methods besides HiCExplorer as presented in the main text: TADbit, TopDom, and Insulation Score. Using each of these methods, we computed TAD boundaries at 0, 1, 4, and 24 hours after TCR-activation. Similar to the TADs inferred by HiCExplorer, the total number of TADs remains relatively constant across multiple time points and that larger Hi-C matrix bin sizes resulted in fewer TADs (**Supplementary Figure S2A**). Whereas the TopDom and Insulation Score methods agree with HiCExplorer in that the number of TADs slightly increases by 24 hours after activation, TADbit demonstrated a slight decrease in the number of TADs. These patterns in the total number of TADs further reflect differences in the median TAD sizes observed under each condition: TAD sizes remained largely unchanged over time post-activation with only a slight decrease in median TAD size observed by 24 hours using both Top Dom (**Supplementary Figure S2B**) and Insulation Score (**Supplementary Figure S2C**). Overall, both of these methods computed TAD boundaries similar to those observed using HiCExplorer in the main text.

Supplementary Figure S2

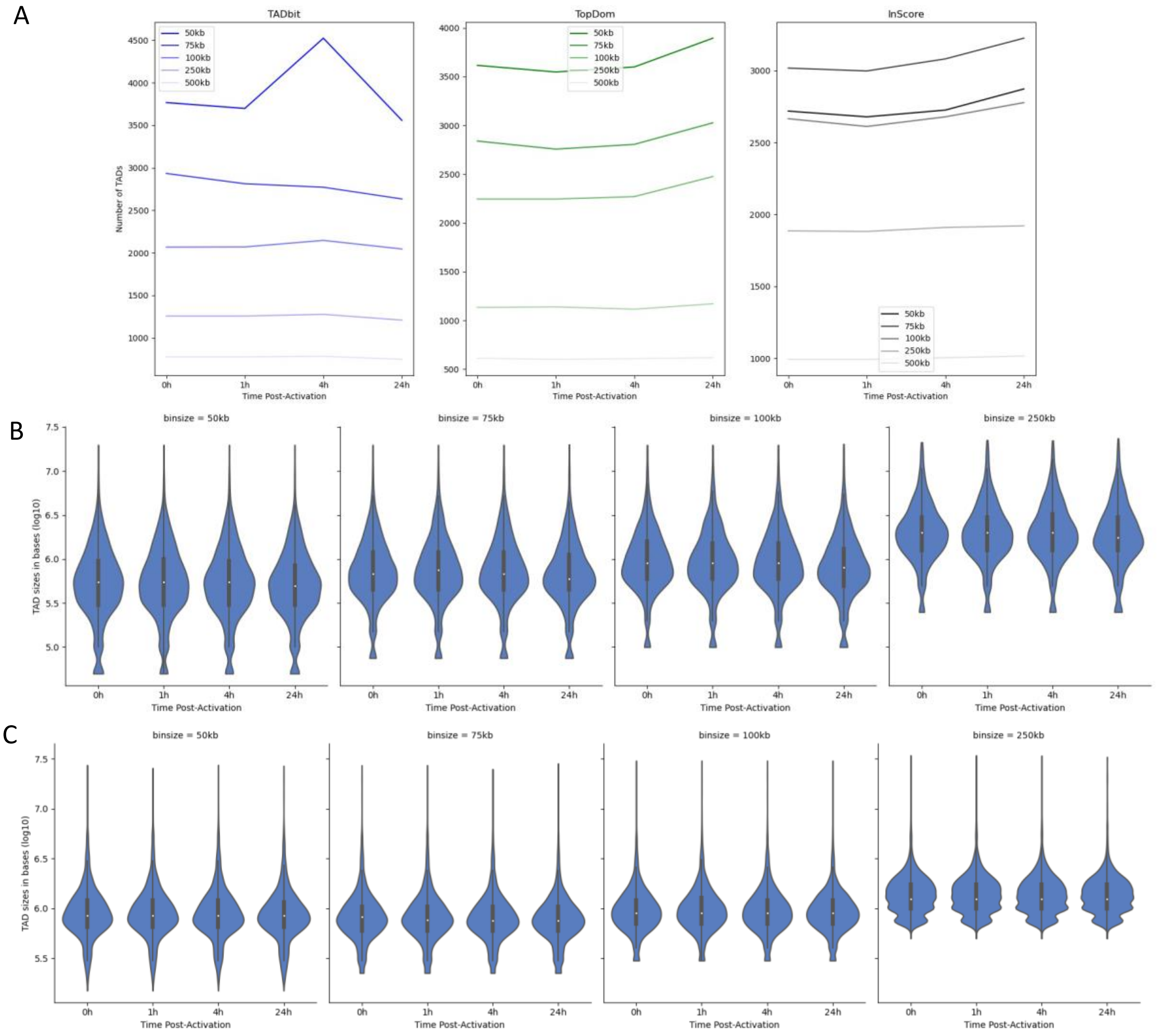

Supplementary Figure S2 – Measuring robustness of TAD calling using alternative TAD-calling algorithms

A) Number of TADs detected using different algorithms (TADbit, TopDom, and Insulation Score) run at different resolutions at 0, 1, 4, and 24 hours after TCR activation.
B) Violin plots depicting the distribution of TAD sizes detected using TopDom at different resolutions (bin sizes) and at 0, 1, 4, and 24 hours after TCR activation.
C) Violin plots depicting distribution of TAD sizes detected using Insulation Score for the same resolutions and hours after TCR activation as shown in panel b.

9.5 Representative Example of TAD Calling by HiCExplorer

Our TADs computed by HiCExplorer spanned a range of sizes. A representative example of TAD calls on chromosome 5 from 126.2 to 150.1 Mb in unstimulated $CD4^+$ T cells demonstrates the concordance of our computed TAD boundaries, as determined by the TAD separation score, with gene localization data and principal components 1 (PC1) and 2 (PC2) from linear decomposition of the constructed Hi-C contact matrices (**Supplementary Figure S3**). Overall, the TADs called by HiCExplorer outlined regions of high Hi-C contact frequency, with boundaries called at local maxima (see arrows) in the TAD separation score. On visual inspection, the computed TAD boundaries often coincided with sign changes in PC1, which generally tracks regions belonging to active versus inactive compartments of chromatin (Lieberman-Aiden, et al., 2009), and in PC2, which generally describes the location along the chromosome (e.g., telomeric vs. centromeric, or on different chromosome arms). Finally, we observed that genes were typically underrepresented within inactive compartments (regions with positive PC1) and more commonly located within active compartments (regions with negative PC1), rather than being uniformly distributed among the computed TADs.

Supplementary Figure S3

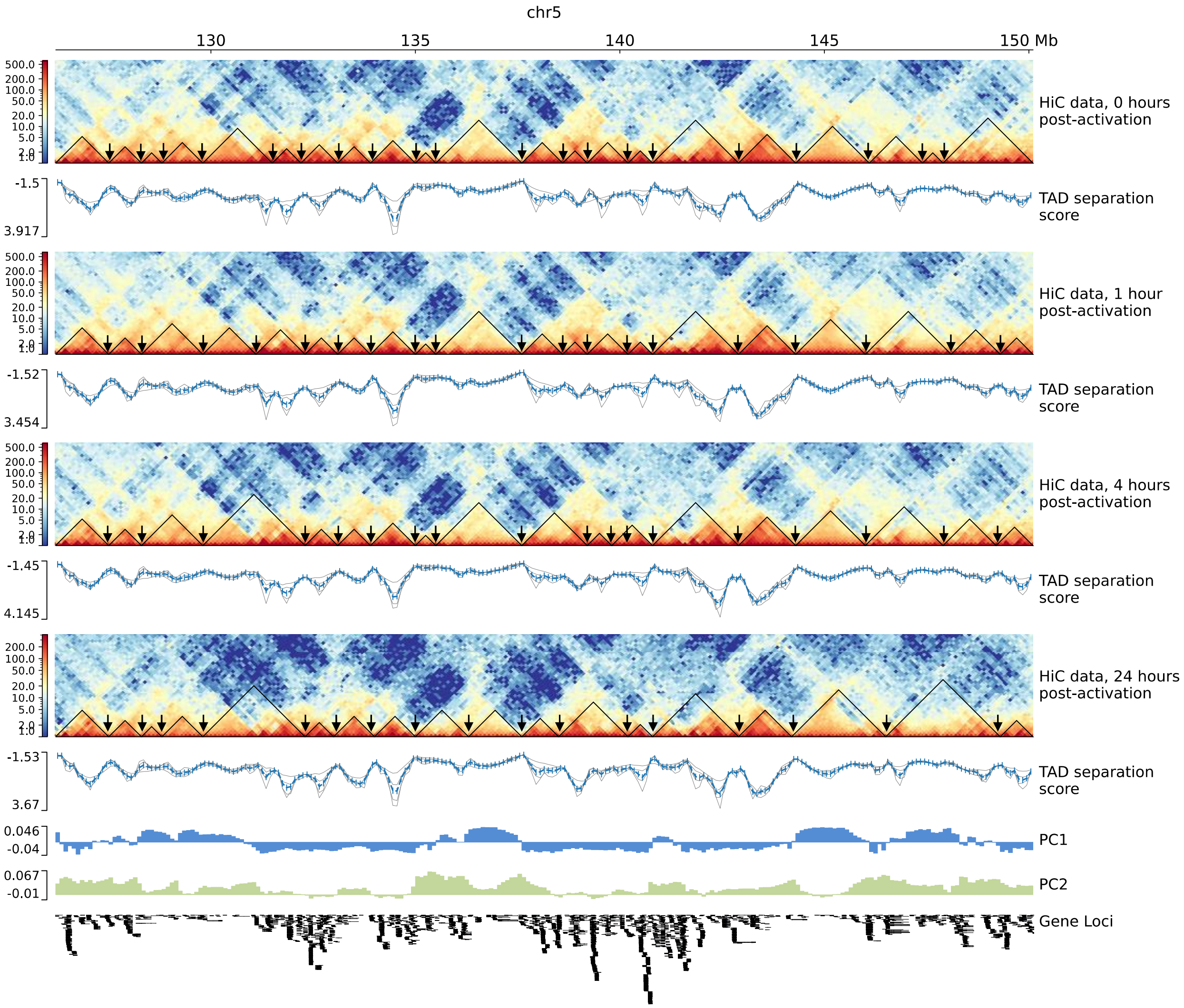

Supplementary Figure S3 – Hi-C Contact Matrices

A representative region (chromosome 5: 126.2 Mb – 150.1 Mb) of the ICE-normalized Hi-C contact matrices for CD4$^+$ T cells at 0, 1, 4, and 24 hours after TCR activation, along with TAD boundary separation scores generated by HiCExplorer at 100 kb. TADs are traced in over each contact matrix. Shown at the bottom are data tracks for:

i. Principal component 1, which generally tracks chromatin active (negative) and inactive (positive) compartments, at 24 hours after TCR activation.
ii. Principal component 2, which generally tracks chromosome location (positive for p-arm, negative for q-arm), at 24 hours after TCR activation.
iii. ENSEMBLE gene loci within the plotted genomic region.

9.6 Varying DEGEF Hyperparameters to Examine Changes in Identified Clusters

We characterized the effects of varying DEGEF's hyperparameters, including window width and different scoring metrics ("count" versus "significance" versus "foldchange"). We tested window widths from 100 kb to 2.5 Mb and compared the resulting FDR profiles on chromosome 11 and chromosome 13 from our main dataset at 24 hours after TCR activation (**Supplementary Figure S4A**). While using the "significance" and "foldchange" scoring metrics (middle and right panels, respectively) resulted in rather similar FDR profiles identifying clusters of upregulated (top panels, red) and of downregulated genes (bottom panels, blue), "count" scoring (left panels) resulted in more stochastic FDR profiles that contain more peaks overall. Additionally, at smaller window sizes (e.g., 100 kb and 250 kb), each window tends to be underpowered to detect significant enrichment, resulting in diminished ability to detect clusters of DEGs surpassing statistical significance. Conversely, at larger window sizes (e.g., 2.5 Mb), statistical power was increased as more genes are encompassed by each window; however, the increased width led to coarser resolution and inclusion of non-cluster genes into the window and thus the DEGEF-called cluster, leading again to diminished ability to detect clusters of DEGs. DEGEF's ability to detect clusters of DEGs appears to be optimized at a midpoint between the extremes of our analysis.

We analyzed the overlap of genes called within peaks in each of the different runs (**Supplementary Figure S4B**). Overall, more similar window sizes resulted in more similar within-cluster-DEGs called. Smaller window sizes displayed greater specificity in identifying cluster genes, as genes identified at a smaller window size had a greater chance of also being identified at a larger window size than vice versa. We also analyzed the total numbers of upregulated and downregulated peaks called under

each combination of window size and scoring metric (**Supplementary Figure S4C**). Interestingly, increasing window size correlated with decreased total number of clusters of DEGs when using the “count” metric, whereas it correlated with increased total number of clusters of DEGs using the ”significance” and “foldchange” metrics. Notably, the “foldchange” metric maximized the number of clusters of upregulated genes at a window size of 750 kb and downregulated genes at a window size of 1 Mb. These observations corroborate our observations about the issues of selecting window-sizes that are either too small or too large.

Supplementary Figure S4

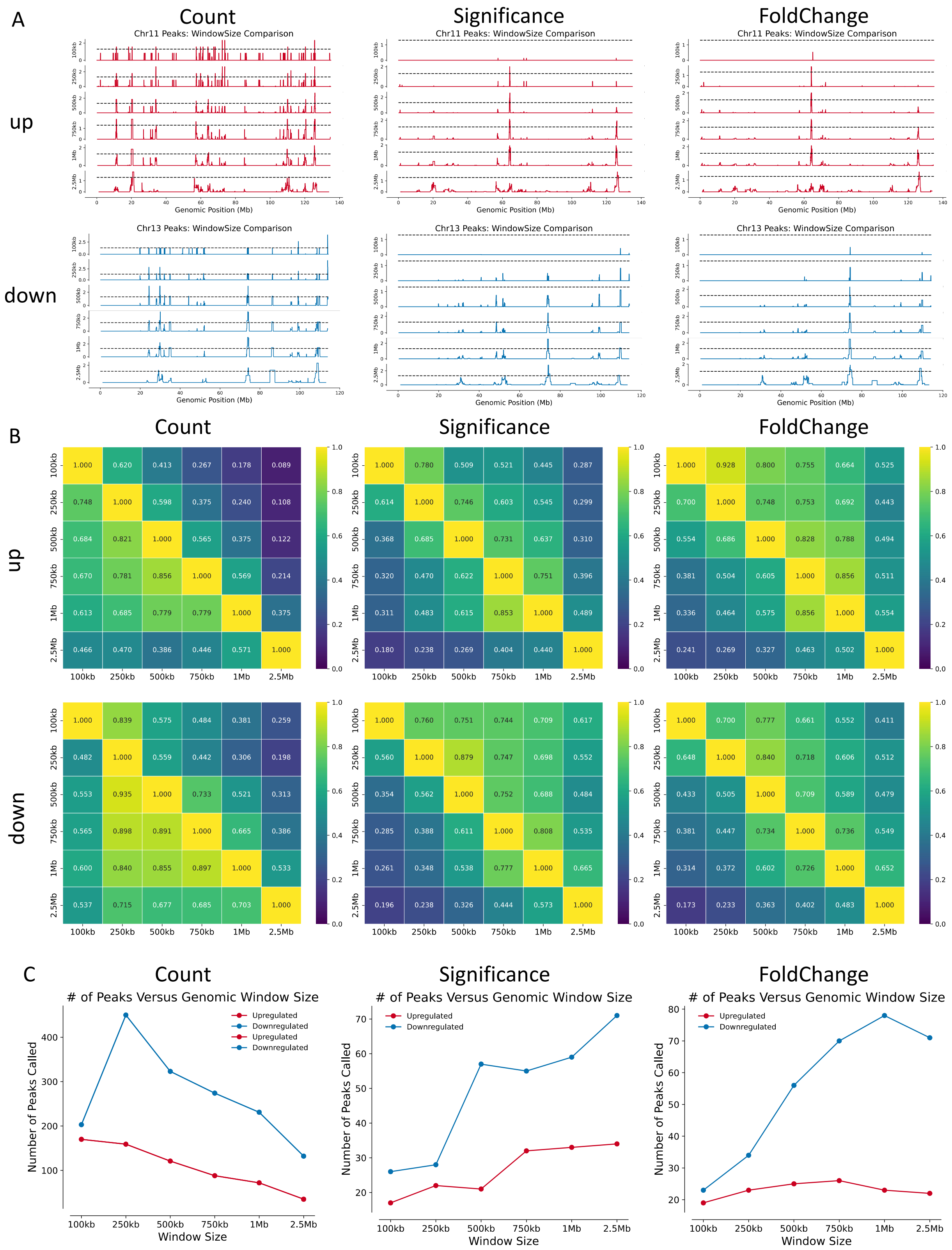

Supplementary Figure S4 – DEGEF Robustness to Window Size Parameter

A) Examples of plots of DEGEF peak-calling using different window sizes, different raw scoring metrics, and either upregulated or downregulated modes using chromosome 11 (top panels) or 13 (bottom panels), respectively, to illustrate the effects of varying each of these parameters in upregulated versus downregulated mode.
B) Matrices demonstrating the robustness of DEGEF in calling genes within enrichment peaks against the window size parameter. Each square represents the proportion of in-cluster genes discovered using the window-size parameter on the y-axis that were also discovered using the window-size parameter on the x-axis (e.g., the proportion of genes in clusters called with WS=1 Mb that were also present in clusters called with WS=750 kb). Matrices were computed for three different raw score modes—“count” (left), “significance” (center), and “foldchange” (right)—and in both “upregulated” (top matrices) and “downregulated” (bottom matrices) modes.
C) Total number of peaks of upregulated genes (red) and downregulated genes (blue) called by DEGEF vs window size parameter at 24 hours post-activation. Trends were computed for three raw score modes (“count,” “significance,” and “foldchange”).

9.7 Central Limit Theorem (CLT) Approximation of Bootstrapped Distributions of Enrichment Scores

To characterize the effectiveness of using the CLT to approximate the bootstrapped null distribution, we compared 4 bootstrapped distributions with their corresponding CLT-approximated “analytic” solutions. To build each of the 4 numerical distributions, 1000 bootstraps were taken using 1, 5, 10, and 25 genes respectively. On visual comparison, whereas the numerical distribution and the CLT-approximated analytic distribution were different for n = 1 and n = 5, the distributions were much more concordant at n = 10 and n = 25 (**Supplementary Figure S5A**). As further evidence of this convergence as the number of genes within the window increases, the enrichment score at which we attain significance at the $\alpha = 0.05$ level decreased and the difference between the enrichment scores also diminished as the number of genes increased (**Supplementary Figure S5B**). We finally also visualized this effect by observing the

difference in the empirical versus CLT-estimated distributions' probability density functions (**Supplementary Figure S5C**). The CLT estimate tended to under-estimate the probability density of bootstrapped scores at lower and higher enrichment scores and over-estimate the probability density of scores at intermediate enrichment scores. However, the magnitude of these over- and under-estimations decreased with increasing numbers of genes in each window.

Supplementary Figure S5

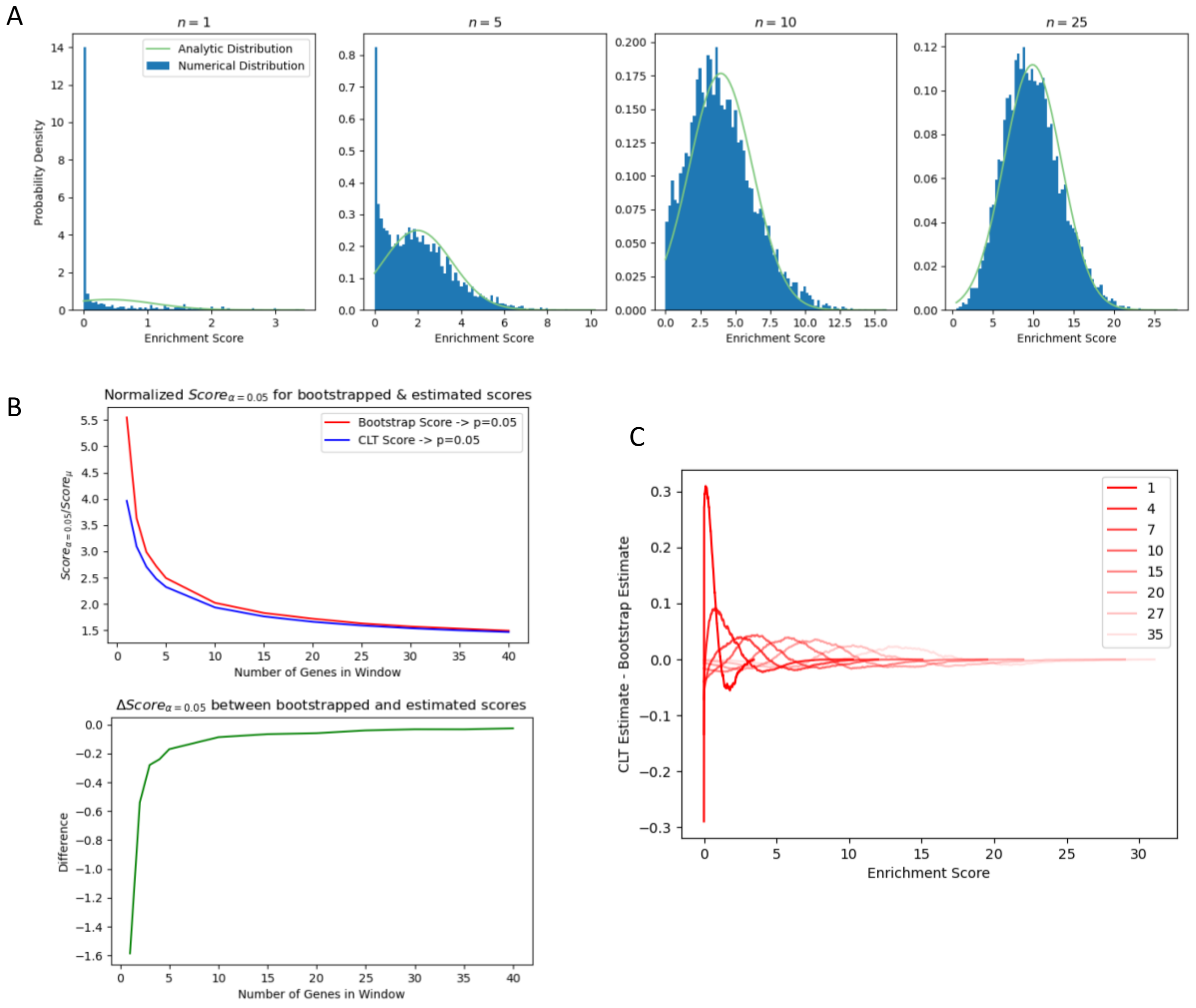

Supplementary Figure S5 – Central Limit Theorem (CLT) can be used in DEGEF Modeling

A) Using the CLT to approximate the null distribution becomes more appropriate as more genes are present in the genomic window. Random bootstrapping enrichment scores for window sizes of 1, 5, 10, and 25 genes demonstrate that

with increasing window size, the numerically simulated null distributions (blue) are progressively better approximated by the CLT estimate of the null distribution (green).

B) Upper panel: The enrichment score (normalized by the average enrichment score) at which $\alpha = 0.05$ according to empirical bootstrapping (red) and analytic estimate using the CLT (blue) for different numbers of genes present in the genomic window (see the Methods for details). The CLT tends to give lower estimates of the enrichment score needed to call significance compared to bootstrapping, but the difference between these scores (lower panel) approaches 0 as the number of genes per genomic window increases.

C) Shown is the difference between the probability mass functions of the empirical bootstrapped null distribution and the CLT estimate of the null distribution at different numbers of genes within the genomic window. As the number of genes in the genomic window increases, the difference between the CLT estimate and the empiric distribution approaches 0.

References

Lieberman-Aiden, E.*, et al.* Comprehensive mapping of long-range interactions reveals folding principles of the human genome. *Science* 2009;326(5950):289-293.

Zhao, S.*, et al.* Comparison of RNA-Seq and microarray in transcriptome profiling of activated T cells. *PLoS One* 2014;9(1):e78644.
